# Serum NO₃⁻ and NO₂⁻ levels among pregnant women from agricultural communities: associations with self-reported dietary and environmental exposures

**DOI:** 10.1038/s41598-026-61462-1

**Published:** 2026-07-10

**Authors:** Fayez A. Abdulla, Yousef Khader, Omar Khabour, Saed Abulubad, Muna Abu-Dalo, Rami Saadeh, Wael K. Al-Delaimy

**Affiliations:** 1 Civil Engineering Department, Jordan University of Science and Technology, Irbid, 22110 Jordan; 2 Department of Public Health, Faculty of Medicine, Jordan University of Science and Technology, Irbid, Jordan; 3 Department of Medical Laboratory Sciences, Faculty of Applied Medical Sciences, Jordan University of Science and Technology, Irbid, Jordan; 4 Chemistry Department, Jordan University of Science and Technology, Irbid, 22110 Jordan; 5 Herbert Wertheim School of Public Health and Human Longevity Science, UC San Diego, 9500 Gilman Dr., La Jolla, CA 92093 USA

**Keywords:** NO₃⁻, NO₂⁻, Pregnancy, Jordan Valley, Dietary exposure, Biomarkers, Diseases, Environmental sciences, Health care, Medical research, Risk factors

## Abstract

Nitrate (NO₃⁻) and nitrite (NO₂⁻) exposure can lead to adverse health impacts, yet data on direct biomarkers are limited. To assess serum NO₃⁻ and NO₂⁻ levels among pregnant women in two agricultural regions of the Jordan Valley in relation to dietary and environmental factors. This cross-sectional study recruited 346 pregnant women aged 18 years or older from four hospitals in the Ghor region between 2023 and 2024. Serum NO₃⁻ and NO₂⁻ levels were measured using a Griess assay. Socio-demographic data, dietary habits, and water source information were gathered through questionnaires. The median serum NO₂⁻ level was 0.63 µM and the median serum NO₃⁻ level was 19.13 µM in the overall sample. Serum NO₂⁻ was significantly higher among women from North Ghor compared to South Ghor (0.83 µM vs. 0.45 µM, *p* < 0.001), while serum NO₃⁻ levels did not significantly differ between regions. In multivariable models adjusting for maternal age, BMI, hypertension, anemia, and smoking, we found that variables of region of residency and eating green beans once a week or more were significantly associated with higher NO₂⁻ levels in the whole sample (*p* < 0.001 and *p* = 0.031, respectively) and in North Ghor (*p* = 0.032), while hypertension was associated with lower NO₂⁻ levels (*p* = 0.032 whole sample; *p* = 0.013 North Ghor). For NO₃⁻, smoking during pregnancy was associated with lower levels in the whole sample (*p* = 0.003) and in South Ghor (*p* = 0.004), while employment in crop harvesting/collection was associated with higher NO₃⁻ levels in South Ghor (*p* = 0.024). Regional differences in serum NO₂⁻ levels exist among pregnant women in rural Jordan, influenced primarily by dietary habits, while occupational exposure to crop harvesting and smoking were associated with serum NO₃⁻ levels. Targeted recommendations and public health strategies are needed to mitigate NO₃⁻ and NO₂⁻ exposure.

## Introduction

Nitrate (NO₃⁻) and nitrites (NO₂⁻) are nitrogenous compounds that naturally occur in the environment and are widely utilized in agriculture and food preservation. NO₃⁻ and NO₂⁻ are commonly found in vegetables, drinking water (particularly groundwater in agricultural areas), and processed meats. Among these compounds, NO₃⁻ is more stable and, therefore, more frequently ingested^1^.

The primary route of human exposure to NO₃⁻ and NO₂⁻ is through dietary intake. Leafy green vegetables, such as spinach and lettuce, are particularly rich in naturally occurring NO₃⁻, while NO₂⁻ is more commonly consumed through processed and cured meats, where it is used as a preservative^2^. In many regions, especially those dependent on private wells or situated in agricultural areas, drinking water contaminated with NO₃⁻ poses a significant exposure risk^1,3,4^.

In the Middle East and North Africa (MENA) region, including Jordan, groundwater is heavily utilized for drinking, particularly in rural communities^5^. Agricultural runoff containing nitrogen-based fertilizers significantly contributes to NO₃⁻ contamination in groundwater. This problem is exacerbated by the combination of poorly regulated fertilizer application and insufficient wastewater treatment, which is common in countries with intensive farming practices and limited environmental oversight^6^.

NO₃⁻ exposure varies based on geographical location, socioeconomic status, and infrastructure availability. Rural areas often lack access to centralized water supplies, relying instead on private wells, which are more prone to contamination. NO₃⁻ contamination in groundwater has been reported in many countries in MENA^7–9^. Additionally, lower-income households may have limited access to purified or bottled water and are more likely to consume lower-cost processed foods containing NO₂⁻ preservatives. These disparities result in higher exposure levels among socioeconomically disadvantaged groups. NO₃⁻ levels in water often peak after fertilizer application seasons and during periods of heavy rainfall due to runoff and leaching.

Once ingested, NO₃⁻ is absorbed in the upper gastrointestinal tract and partially secreted in saliva, where oral bacteria convert it to NO₂⁻. Subsequently, NO₂⁻ can be reduced to nitric oxide (NO), a potent vasodilator involved in blood pressure regulation, endothelial function, and immune responses^1,10^. However, in the acidic environment of the stomach, NO₂⁻ can also react with amines and amides to form N-nitroso compounds (NOCs), such as nitrosamines, many of which are classified as carcinogenic and teratogenic^11^. The risk of endogenous nitrosation increases with high NO₃⁻/NO₂⁻ intake, particularly when accompanied by low antioxidant intake.

Research has explored the potential associations between maternal NO₃⁻/NO₂⁻ exposure and adverse pregnancy outcomes, including intrauterine growth restriction (IUGR) and preterm birth^12^. However, the findings have been inconsistent, largely due to variations in exposure levels, timing, and the source. The most well-established risk associated with excessive NO₃⁻ exposure during pregnancy or infancy is infant methemoglobinemia.

Despite the recognized health implications, there is a paucity of data on direct biomarkers of NO₃⁻ and NO₂⁻ levels in pregnant women, particularly in low- and middle-income countries. Most available studies estimate exposure based on dietary or water intake rather than direct biological measurements. Furthermore, the determinants of NO₃⁻/NO₂⁻ exposure during pregnancy remain poorly characterized^13^. Understanding these factors is crucial, as they may influence both exposure levels and pregnancy outcomes. Given the biological plausibility and the gaps in previous research, it is essential to assess maternal serum NO₃⁻ and NO₂⁻ concentrations to document the levels of exposure and potential risks. This study had the aim to assess NO₃⁻ and NO₂⁻ levels in the blood of pregnant women and determine factors associated with these levels, including socio-demographic factors, dietary patterns, and water sources.

## Methods

### Study design and sampling

**Figure 1:**
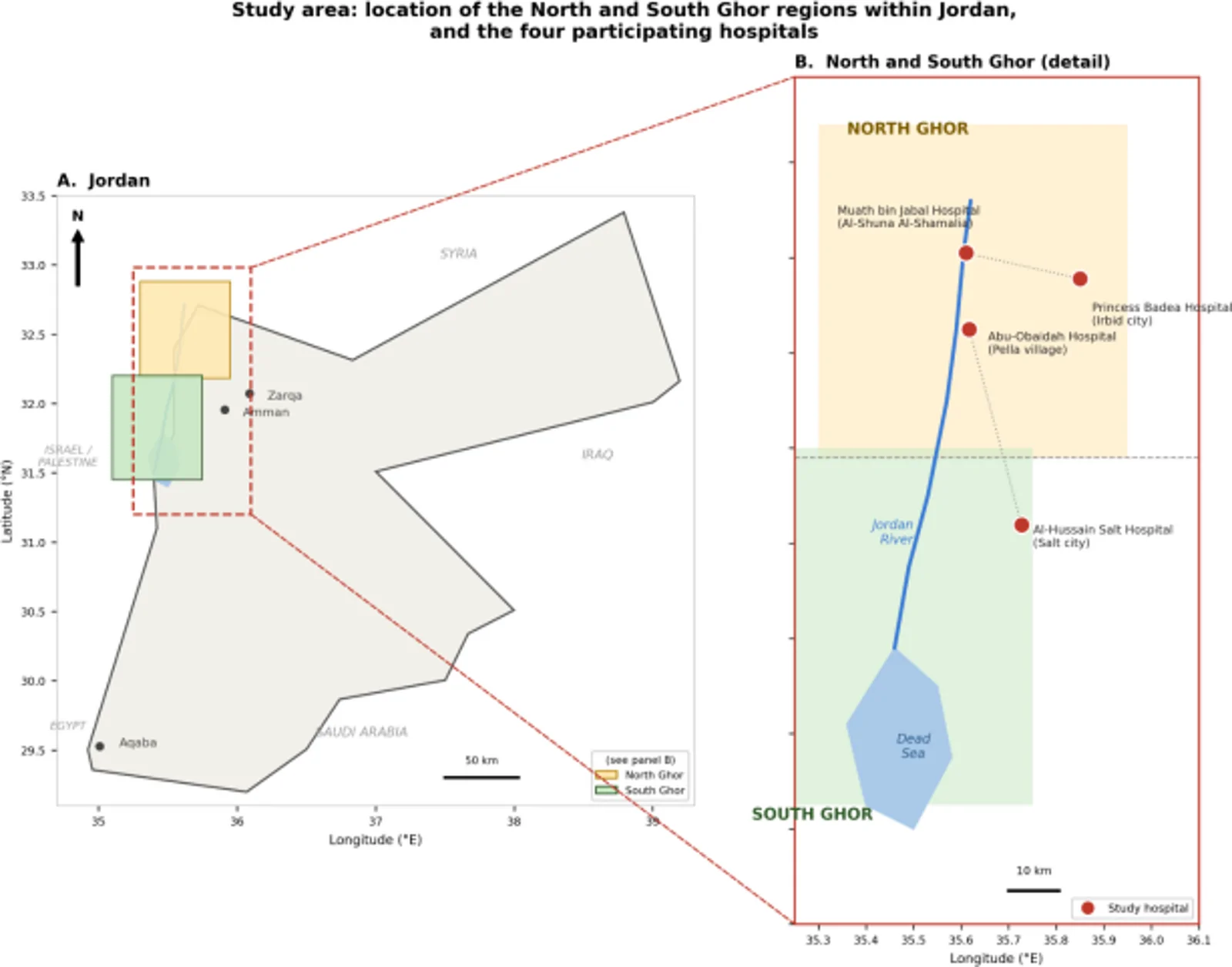
(**A**) Map of Jordan showing the location of the North and South Ghor regions within the Jordan Valley relative to the whole country; (**B**) detailed view of the North and South Ghor catchment areas, the Jordan River and Dead Sea, and the precise locations of the four participating hospitals. The map is illustrative and not drawn to a precise GIS scale.

This was a cross-sectional study aimed to evaluate serum NO₂⁻ and NO₃⁻ levels among pregnant women residing in the North and South Ghor regions of the Jordan Valley who were admitted for delivery. A systematic random sampling method was utilized to select pregnant Jordanian women aged 18 years or older who presented for delivery between 2023 and 2024 at four primary hospitals serving the Ghor region: Muath bin Jabal Hospital (Alshuna Alshamalia), Abu-Obaidah Hospital (Pella village), Princess Badea Hospital (Irbid city), and AlHussain Salt Hospital (Salt city). Figure 1 shows the locations of the four study hospitals relative to the North and South Ghor catchment areas in the Jordan Valley; participants were classified as residing in North or South Ghor based on self-reported place of residence rather than hospital of delivery, since women from either region could deliver at any of the four hospitals depending on accessibility.

Inclusion criteria were limited to women who reported their residence in either the North or South Ghor regions as areas of high agricultural activities. Women with chronic medical conditions (e.g., cardiovascular disease, cancer, autoimmune disorders, blood disorders, metabolic diseases, kidney diseases, and endocrine disorders) were excluded. Additionally, women who experienced adverse pregnancy outcomes after the index delivery, such as preterm birth, miscarriage, or intrauterine fetal death, were also excluded. This selective inclusion criterion ensured a focus on apparently healthy pregnant women.

### Sample size

The sample size was calculated using G*Power software to determine the minimum number of participants needed to detect statistically significant differences in serum NO₂⁻ and NO₃⁻ levels across subgroups. Assuming a moderate effect size (Cohen’s d = 0.4), an alpha level of 0.05, and 80% statistical power, the minimum required sample size was calculated to be 200 participants. The actual study sample consisted of 346 pregnant women, with 199 from the North Ghor region and 147 from the South Ghor region, thereby exceeding the calculated minimum sample size. This larger sample size not only provided robust statistical power to detect regional differences in serum NO₂⁻ and NO₃⁻ concentrations but also allowed for the assessment of associations between regional dietary and environmental exposures and NO₂⁻/NO₃⁻ levels.

### Questionnaire

A structured questionnaire was developed to capture comprehensive information from participants prior to hospital discharge. While blood samples were collected upon admission, questionnaire data were collected after delivery and before discharge, to ensure participant comfort and avoid any bias related to the stress of labor. The questionnaire was designed to gather socio-demographic information and details about dietary habits, water sources, and potential environmental exposures. Key socio-demographic variables included age, body mass index (BMI), employment status, place of residence, household income, and education level. Additionally, medical history variables were collected, including anemia, fever, urinary tract infections, and hypertension, all referring to conditions occurring during the index pregnancy and self-reported by participants prior to hospital discharge.

Dietary exposure data focused on the consumption of NO₃⁻-rich foods, particularly root vegetables and leafy greens (e.g., beetroot, spinach, parsley, watercress, and cabbage). Participants were asked about the frequency of their consumption of these vegetables. They were also asked about local farming practices and pesticide use. Data were also gathered on water sources, including municipal, rainwater, and filtered water, and whether participants were involved in farming activities or used local water sources for drinking and cooking. We note that, throughout this manuscript, “environmental exposure” refers to self-reported water source, farming activity, and pesticide-use variables rather than direct chemical measurement of NO₃⁻/NO₂⁻ in environmental media. The questionnaire was developed by the study team, informed by instruments used in prior NO₃⁻ exposure studies, and reviewed by the team for face and content validity; formal psychometric validation was not conducted.

### Blood sampling

Whole blood samples (4 mL) were collected from each participant using pro-coagulation plain tubes upon admission to the hospital for delivery. The samples were allowed to clot at room temperature for 30 min and then centrifuged (Sigma 1‑7, Germany) at 1500 × g for 10 min to separate the serum. Serum was then ultra-filtered through a 10 kDa molecular weight cutoff filter (Merck Millipore, Germany) and centrifuged at 16,000 rpm (DLAB D3024R, MediLab Tech, Los Angeles, CA, USA). The serum was transferred into 1.5 mL tubes and stored at -80˚C until further analysis. This method ensured the preservation of serum components for subsequent NO₂⁻/NO₃⁻ analysis.

### Serum NO₂⁻/NO₃⁻ assay

Serum NO₂⁻ and NO₃⁻ levels were measured colorimetrically using a commercially available kit from Cayman Chemical Company (Ann Arbor, MI, USA). The assay utilized Griess reagents, a standard method for measuring NO₂⁻ and NO₃⁻, and total NO₃⁻/NO₂⁻ concentrations. Serum samples were diluted three times with assay buffer as per kit instructions. Total NO₃⁻/NO₂⁻ levels were measured after treatment with NO₃⁻ reductase enzyme, while NO₂⁻ was quantified separately in the absence of the enzyme. NO₃⁻ levels were derived by subtracting NO₂⁻ concentrations from total NO₃⁻/NO₂⁻ values. The assays were performed in duplicate to ensure accuracy, and absorbance changes were measured at 540 nm using a Bio-Tek plate reader (Winooski, USA).

### Ethical approval

The study received ethical approval from the Institutional Review Board (IRB) at King Abdullah University Hospital and Jordan University of Science and Technology (ID number: 47/151/2022). Informed written consent was obtained from all participants in accordance with the ethical standards outlined by the IRB, ensuring their autonomy and confidentiality throughout the study process.

### Statistical analysis

Data were analyzed using SPSS version 24 (IBM Corp., Armonk, NY). Continuous variables were summarized as means (SD) and medians. Differences in sociodemographic and clinical characteristics between women from the North and South Ghor regions were tested using chi-square tests for categorical variables and the Mann–Whitney U test for continuous variables with non-normal distributions, including NO₂⁻ and NO₃⁻ levels. For comparisons among more than two groups, the Kruskal–Wallis H test was used. Prior to multivariate analysis, serum NO₂⁻ and NO₃⁻ levels were log-transformed to normalize their distribution. Multivariate linear regression models were constructed to identify predictors of serum NO₂⁻ and NO₃⁻ concentrations, both for the overall sample and stratified by region. We used a forced-entry method, knowledge-based multivariable approach: maternal age, BMI, hypertension, anemia, and smoking status were entered as core covariates in every model, region was additionally entered in the whole-sample models, and green bean, chard, and radish consumption, municipal water use for irrigation, and employment in crop harvesting/collection were entered simultaneously as the dietary/environmental exposures of interest. No variables were added to or removed from the models based on statistical significance. Results were reported as regression coefficients (B), 95% confidence intervals (CIs), and p-values. A significance threshold of *p* < 0.05 was applied for all statistical tests.

## Results

### Participants’ characteristics and regional dietary differences

This study included 346 women (199 from North Ghor and 147 from South Ghor), with a mean (SD) age of 29.3 (6.1) years. Nearly half of the participants (52.4%) were aged between 26 and 35 years. Table 1 shows the socio-demographic characteristics, water source use, and dietary consumption patterns of women in North and South Ghor.

**Table 1:** Socio-demographic characteristics, water source use, and dietary consumption patterns of women in North and South Ghor.

Variable	North Ghor	South Ghor	Total*	*P*-value
Mother’s Age				0.369
≤ 25	66 (34.2%)	39 (27.7%)	105 (31.4%)	
26–35	99 (51.3%)	76 (53.9%)	175 (52.4%)	
> 35	28 (14.5%)	26 (18.4%)	54 (16.2%)	
Mother’s education				0.983
High school or less	168 (84.8%)	124 (84.9%)	292 (84.9%)	
Diploma or higher	30 (15.2%)	22 (15.1%)	52 (15.1%)	
Mother’s occupation				0.582
Housewife	180 (91.4%)	133 (93.0%)	313 (92.1%)	
Employed	17 (8.6%)	10 (7.0%)	27 (7.9%)	
Income (Jordanian dinars per month)				0.002
≤ 200	40 (22.3%)	21 (14.4%)	61 (18.5%)	
201–300	71 (39.7%)	86 (58.9%)	157 (48.3%)	
> 300	68 (38.0%)	39 (26.7%)	107 (32.9%)	
Smoking during pregnancy	18 (9.0%)	8 (5.3%)	26 (7.4%)	0.191
Anemia	91 (46.0%)	53 (35.8%)	144 (41.6%)	0.058
Hypertension	8 (4.0%)	10 (6.8%)	18 (5.1%)	0.242
Source of water				
Municipal water for drinking	49 (24.3%)	25 (16.7%)	74 (21.0%)	0.084
Municipal water for cooking	148 (73.3%)	114 (76.0%)	262 (74.4%)	0.561
Municipal water for irrigation	158 (78.2%)	108 (72.0%)	266 (75.6%)	0.179
Filtered water for drinking	157 (77.7%)	120 (80.0%)	277 (78.7%)	0.606
Filtered water for cooking	43 (21.3%)	33 (22.0%)	76 (21.6%)	0.872
Employment in crop harvesting/collection	27 (13.4%)	11 (7.3%)	38 (10.8%)	0.071
Eating fruits without washing	30 (14.9%)	10 (6.7%)	40 (11.4%)	0.017
Food consumption once weekly or more				
Arugula	53 (26.2%)	84 (56.0%)	137 (38.9%)	< 0.001
Chard	43 (21.3%)	15 (10.0%)	58 (16.5%)	0.005
Spinach	103 (51.0%)	68 (45.3%)	171 (48.6%)	0.294
Parsley	158 (78.2%)	127 (84.7%)	285 (81.0%)	0.127
Cabbage	148 (73.3%)	66 (44.0%)	214 (60.8%)	< 0.001
Fenugreek	78 (38.6%)	25 (16.7%)	103 (29.3%)	< 0.001
Beet Roots	18 (8.9%)	10 (6.7%)	28 (8.0%)	0.442
Celery	62 (30.7%)	6 (4.0%)	68 (19.3%)	< 0.001
Radishes	125 (61.9%)	53 (35.3%)	178 (50.6%)	< 0.001
Turnips	125 (61.9%)	32 (21.3%)	157 (44.6%)	< 0.001
Cauliflower	151 (74.8%)	128 (85.3%)	279 (79.3%)	0.015
Eggplant	163 (80.7%)	132 (88.0%)	295 (83.8%)	0.066
Green Beans	136 (67.3%)	103 (68.7%)	239 (67.9%)	0.790

*The totals across variables vary due to missing data in some variables. Anemia, hypertension, and income are self-reported. “Chard” (local term سلق) refers to Swiss chard/leaf beet (Beta vulgaris subsp. vulgaris var. cicla) and is distinct from “Beet Roots” (local term شمندر), which refers to table/sugar beet (Beta vulgaris subsp. vulgaris var. conditiva).

The majority (84.9%) had a high school education or less, while 15.1% had attained a diploma or higher qualification. Most mothers were housewives (92.1%), with only 7.9% were employed. Almost half (48.3%) of participants reported a monthly household income earning between 201 and 300 JD, 32.9% reported earning more than 300 JD, and 18.5% reported earning 200 JD or less. Significant regional differences were observed in income and consumption of selected food items between women from North and South Ghor (Table 1). A higher proportion of women in South Ghor fell into the lower 201–300 income category compared to North Ghor (58.9% vs. 39.7%, *p* = 0.002). Consumption of arugula was significantly higher among women in South Ghor than among women in North Ghor (56.0% vs. 26.2%, *p* < 0.001), as was the consumption of cauliflower (85.3% vs. 74.8%, *p* = 0.015). The intake of cabbage was higher in North Ghor (73.3% vs. 44.0%, *p* < 0.001), as was the consumption of fenugreek (38.6% vs. 16.7%, *p* < 0.001), chard (21.3% vs. 10.0%, *p* = 0.005), celery (30.7% vs. 4.0%, *p* < 0.001), radishes (61.9% vs. 35.3%, *p* < 0.001), and turnips (61.9% vs. 21.3%, *p* < 0.001).

### Serum NO₂⁻ and NO₃⁻ levels among women in North and South Ghor

The median serum NO₂⁻ level in the overall sample of women was 0.63 µM, and the median NO₃⁻ level was 19.13 µM (Table 2). Women residing in North Ghor had significantly higher median serum NO₂⁻ levels compared to those in South Ghor (0.83 µM vs. 0.45 µM, *p* < 0.001). However, no statistically significant difference was observed in median serum NO₃⁻ levels between women in North and South Ghor (19.71 µM vs. 17.96 µM, *p* = 0.105).

**Table 2:** Serum NO₂⁻ and NO₃⁻ among women in North Ghor and South Ghor.

Residency	NO₂⁻ (uM)			NO₃⁻ (uM)		
	Mean (SD)	Median	*P*-value*	Mean	Median	*P*-value *
North Ghor	1.01 (0.65)	0.83	< 0.001	22.10 (11.21)	19.71	0.105
South Ghor	0.60 (0.48)	0.45		22.39 (17.23)	17.96	
Total	0.84 (0.62)	0.63		22.22 (14.06)	19.13	

*P-value for median differences (North Ghor vs. South Ghor) using Mann–Whitney U Test.

### Serum NO₂⁻ and NO₃⁻ levels by women’s demographic and clinical characteristics

Tables 3 and 4 show the serum NO₂⁻ and NO₃⁻ levels among women by demographic and clinical characteristics in North Ghor and South Ghor, respectively. In North Ghor, there were significant differences in median serum NO₂⁻ levels across income levels (*p* = 0.016), with women in the higher income groups having higher levels (201–300 JD (0.94 µM) and > 300 JD income groups (0.79 µM)) compared to low income (≤ 200 JD) having the lowest median NO₂⁻ level (0.61 µM). NO₂⁻ levels also differed significantly by anemia status (*p* = 0.049), with women with anemia showing higher median NO₂⁻ levels (0.96 µM) than women without anemia (0.78 µM). No statistically significant differences were observed in median NO₃⁻ levels across any of the assessed variables. Additionally, variables such as age, education, occupation, and smoking during pregnancy did not show significant differences in either NO₂⁻ or NO₃⁻ levels.

**Table 3:** Serum NO₂⁻ and NO₃⁻ among women in North Ghor by demographic and clinical characteristics.

Variable	N	NO₂⁻ (µM)			NO₃⁻ (µM)		
		Mean (SD)	Median	P-value for median differences*	Mean (SD)	Median	P-value for median differences*
Age				0.870			0.370
≤ 25	67	1.06 (0.92)	0.83		22.76 (13.09)	19.10	
26–35	100	1.06 (0.72)	0.83		20.65 (9.16)	19.69	
> 35	29	1.02 (0.72)	0.82		27.43 (18.84)	22.86	
Education				0.433			0.970
High school or less	170	1.01 (0.69)	0.83		22.59 (13.11)	19.41	
Diploma or higher	31	1.29 (1.19)	1.01		21.08 (7.98)	20.48	
Occupation				0.089			0.584
Housewife	183	1.04 (0.80)	0.83		22.51 (12.73)	19.89	
Employed	17	1.22 (0.62)	1.20		20.41 (9.65)	18.78	
Income (Jordanian dinars per month)				0.016			0.993
≤ 200	40	0.78 (0.65)	0.61		21.52 (10.02)	19.89	
201–300	72	1.15 (0.81)	0.94		21.93 (10.57)	20.24	
> 300	69	1.07 (0.87)	0.79		22.40 (12.37)	20.76	
Smoking during pregnancy				0.217			0.695
No	181	1.04 (0.78)	0.83		22.70 (12.83)	19.70	
Yes	18	1.25 (0.87)	1.20		20.49 (9.39)	19.83	
Anemia				0.049			0.984
No	107	1.00 (0.87)	0.78		22.83 (14.2)	19.69	
Yes	91	1.11 (0.70)	0.96		21.92 (10.5)	19.44	

*The Kruskal–Wallis H test was used to compare medians across different age and income categories, while the Mann–Whitney U test was applied to compare medians between categories of other variables.

**Table 4:** Serum NO₂⁻ and NO₃⁻ among women in South Ghor by demographic and clinical characteristics.

Variable	N	NO₂⁻ (µM)			NO₃⁻ (µM)		
		Mean (SD)	Median	P-value for median differences*	Mean (SD)	Median	P-value for median differences*
Age				0.900			0.461
≤ 25	39	0.60 (0.43)	0.41		21.91 (19.73)	13.25	
26–35	79	0.61 (0.50)	0.48		25.30 (21.19)	19.00	
> 35	26	0.64 (0.53)	0.45		20.83 (17.41)	15.40	
Education				0.648			0.116
High school or less	127	0.60 (0.47)	0.45		25.48 (21.68)	18.21	
Diploma or higher	22	0.59 (0.51)	0.36		16.03 (8.15)	16.78	
Occupation				0.070			0.590
Housewife	136	0.63 (0.49)	0.48		24.53 (21.10)	18.05	
Employed	10	0.41 (0.29)	0.29		19.37 (14.29)	15.89	
Income (Jordanian dinars per month)				0.560			0.181
≤ 200	21	0.57 (0.59)	0.37		26.16 (15.22)	23.20	
201–300	88	0.61 (0.48)	0.46		25.19 (22.86)	18.17	
> 300	40	0.62 (0.41)	0.48		20.21 (17.23)	13.51	
Smoking during pregnancy				0.544			0.022
No	142	0.61 (0.49)	0.45		24.76 (20.73)	18.41	
Yes	8	0.47 (0.27)	0.33		10.60 (6.92)	12.30	
Anemia				0.142			0.588
No	95	0.63 (0.45)	0.48		25.52 (22.74)	18.43	
Yes	53	0.55 (0.48)	0.37		21.15 (15.90)	14.50	

* The Kruskal–Wallis H test was used to compare medians across different age and income categories, while the Mann–Whitney U test was applied to compare medians between categories of other variables.

In South Ghor, there was a significant difference in median serum NO₃⁻ levels based on smoking during pregnancy (*p* = 0.022). Women who did not smoke during pregnancy had a higher median NO₃⁻ level (18.41 µM) compared to those who did smoke (12.30 µM). No other statistically significant differences were observed in median serum NO₂⁻ or NO₃⁻ levels across age, education, occupation, income, or anemia status. While there was a borderline non-significant difference in NO₂⁻ levels by occupation (*p* = 0.070), housewives had higher median NO₂⁻ levels (0.48 µM) compared to employed women (0.29 µM).

### Serum NO₂⁻, NO₃⁻ levels by water source and Dietary intake

In the analysis of environmental and dietary factors, most water-source variables were not significantly associated with serum NO₂⁻ or NO₃⁻ levels; however, in South Ghor, non-municipal irrigation water was associated with higher serum NO₃⁻ levels in the unadjusted analysis. In North Ghor (Table 5), women who consumed green beans had significantly higher median serum NO₂⁻ levels compared to non-consumers (0.90 µM vs. 0.78 µM, *p* = 0.007). Similarly, the consumption of chard was associated with significantly higher median serum NO₃⁻ levels (24.02 µM vs. 18.83 µM, *p* = 0.015).

**Table 5:** Serum NO₂⁻ and NO₃⁻ levels among women by water sources and food consumption in North Ghor.

Water Source/Food	N	NO₂⁻ (µM)			NO₃⁻ (µM)		
		Mean (SD)	Median	P-value for median differences	Mean (SD)	Median	P-value for median differences
Using municipal water for drinking				0.749			0.814
No	153	1.07 (0.84)	0.83		22.89 (13.33)	19.70	
Yes	49	1.00 (0.60)	0.83		21.12 (9.44)	20.24	
Using municipal water for cooking				0.647			0.189
No	54	1.06 (0.99)	0.83		25.40 (15.78)	20.70	
Yes	148	1.05 (0.70)	0.83		21.39 (10.93)	19.69	
Using municipal water for irrigation				0.847			0.573
No	44	1.01 (0.71)	0.85		24.50 (16.34)	20.70	
Yes	158	1.06 (0.81)	0.83		21.89 (11.20)	19.65	
Using filtered water for drinking				0.64			0.64
No	45	1.20 (1.11)	0.83		21.29 (10.42)	18.97	
Yes	157	1.01 (0.66)	0.83		22.80 (13.05)	20.08	
Using filtered water for Cooking				0.11			0.293
No	159	1.07 (0.70)	0.83		21.82 (11.55)	19.67	
Yes	43	0.99 (1.05)	0.76		24.82 (15.46)	20.63	
Employment in crop harvesting/collection				0.491			0.605
No	175	1.05 (0.81)	0.83		22.51 (13.07)	19.89	
Yes	27	1.06 (0.64)	0.86		22.12 (8.06)	19.17	
Arugula				0.326			0.251
No	149	1.04 (0.66)	0.83		22.04 (12.62)	19.44	
Yes	53	1.09 (1.07)	0.79		23.64 (12.20)	22.02	
Chard				0.337			0.015
No	159	1.06 (0.73)	0.83		21.87 (13.12)	18.83	
Yes	43	1.01 (0.99)	0.73		24.64 (9.71)	24.02	
Parsley				0.855			0.703
No	44	0.97 (0.51)	0.90		22.75 (11.25)	19.38	
Yes	158	1.07 (0.85)	0.81		22.38 (12.86)	20.08	
Cabbage				0.283			0.802
No	54	0.90 (0.55)	0.83		21.95 (9.44)	20.29	
Yes	148	1.10 (0.85)	0.83		22.65 (13.47)	19.71	
Fenugreek							0.586
No	124	0.99 (0.73)	0.83	0.073	22.16 (12.24)	19.44	
Yes	78	1.15 (0.86)	0.93		22.93 (12.97)	20.12	
Beetroot				0.171			0.233
No	184	1.00 (0.67)	0.83		22.23 (12.55)	19.54	
Yes	18	1.53 (1.48)	1.14		24.77 (12.13)	23.64	
Radishes				0.176			0.748
No	77	0.96 (0.68)	0.83		21.53 (10.35)	19.43	
Yes	125	1.11 (0.84)	0.83		23.03 (13.67)	20.08	
Turnips				0.452			0.982
No	77	1.07 (0.72)	0.88		21.78 (9.89)	19.17	
Yes	125	1.04 (0.83)	0.72		22.88 (13.89)	20.08	
Cauliflower				0.284			0.17
No	51	0.95 (0.67)	0.83		23.63 (10.92)	22.62	
Yes	151	1.08 (0.82)	0.83		22.06 (13.00)	19.44	
Eggplant				0.069			0.42
No	39	0.85 (0.55)	0.72		22.93 (10.81)	22.55	
Yes	163	1.10 (0.83)	0.83		22.35 (12.90)	19.31	
Green Beans				0.007			0.823
No	66	0.88 (0.68)	0.78		22.40 (12.97)	19.07	
Yes	136	1.14 (0.82)	0.90		22.49 (12.32)	20.12	

* Mann–Whitney U test.

Among women in South Ghor (Table 6), serum NO₂⁻ and NO₃⁻ levels showed limited variation by water source, though some differences reached statistical significance. The use of non-municipal water for irrigation (wells, tanks) was significantly associated with higher median serum NO₃⁻ levels (25.26 µM) compared to those using municipal water for irrigation (15.48 µM, *p* = 0.006). Similarly, women working in crop collection had markedly higher serum NO₃⁻ levels (32.35 µM) than non-workers (16.25 µM, *p* = 0.003). For NO₂⁻, a significant difference was observed with those who reported not consuming radish having higher median serum NO₂⁻ levels (0.52 µM) compared to consumers (0.37 µM, *p* = 0.013). Other food and water-related exposures showed no significant differences in serum NO₂⁻ or NO₃⁻ levels.

**Table 6:** Serum NO₂⁻ and NO₃⁻ levels among women by water sources and food consumption in South Ghor.

Water Source/Food	N	NO₂⁻ (µM)			NO₃⁻ (µM)		
		Mean (SD)	Median	P-value for median differences	Mean (SD)	Median	P-value for median differences
Using Municipal Water for Drinking				0.873			0.212
No	125	0.59 (0.43)	0.45		23.79 (21.04)	16.32	
Using Municipal Water for Cooking				0.306			0.700
No	36	0.53 (0.39)	0.45		23.49 (20.55)	14.79	
Yes	114	0.63 (0.50)	0.41		24.16 (20.54)	18.41	
Using Municipal Water for Irrigation				0.462			0.006
No	42	0.67 (0.65)	0.37		26.98 (17.28)	25.26	
Yes	108	0.58 (0.39)	0.48		22.84 (21.55)	15.48	
Using Filtered Water for Drinking				0.614			0.118
No	30	0.73 (0.66)	0.41		29.05 (24.12)	20.68	
Yes	120	0.58 (0.42)	0.45		22.74 (19.37)	16.25	
Using filtered water for Cooking				0.143			0.768
No	114	0.63 (0.50)	0.48		22.10 (17.16)	17.70	
Yes	33	0.52 (0.41)	0.36		23.39 (17.8)	17.96	
Employment in crop harvesting/collection				0.962			0.003
No	136	0.60 (0.47)	0.45		21.6 (17.41)	16.25	
Yes	11	0.66 (0.57)	0.36		31.84 (11.6)	32.35	
Arugula				0.36			0.662
No	66	0.66 (0.51)	0.51		22.40 (18.64)	17.34	
Yes	84	0.57 (0.45)	0.41		25.26 (21.84)	18.01	
Chard				0.56			0.699
No	135	0.62 (0.49)	0.43		23.69 (20.34)	17.96	
Yes	15	0.52 (0.34)	0.49		26.76 (22.16)	20.17	
Parsley				0.596			0.703
No	23	0.73 (0.63)	0.49		26.00 (22.56)	18.41	
Yes	127	0.59 (0.44)	0.41		23.64 (20.15)	17.65	
Cabbage				0.149			0.651
No	84	0.66 (0.51)	0.49		25.11 (23.33)	17.96	
Yes	66	0.54 (0.42)	0.41		22.59 (16.21)	18.99	
Fenugreek				0.615			0.295
No	125	0.59 (0.46)	0.41		24.38 (21.88)	16.32	
Yes	25	0.68 (0.56)	0.63		22.10 (11.18)	21.93	
Beetroot				0.193			0.832
No	140	0.60 (0.48)	0.41		24.40 (21.01)	18.05	
Yes	10	0.68 (0.37)	0.67		18.47 (9.45)	14.92	
Radishes				0.013			0.612
No	97	0.66 (0.48)	0.52		23.33 (18.65)	18.21	
Yes	53	0.52 (0.47)	0.37		25.23 (23.60)	14.33	
Turnips				0.759			0.825
No	118	0.63 (0.52)	0.46		24.26 (21.15)	17.65	
Yes	32	0.54 (0.29)	0.41		23.04 (18.05)	19.43	
Cauliflower				0.164			0.769
No	22	0.72 (0.54)	0.52		26.48 (23.16)	16.21	
Yes	128	0.59 (0.46)	0.41		23.58 (20.05)	18.01	
Eggplant				0.802			0.929
No	18	0.65 (0.58)	0.39		23.29 (17.39)	18.01	
Yes	132	0.60 (0.46)	0.45		24.10 (20.92)	17.96	
Green Beans				0.118			0.746
No	47	0.57 (0.54)	0.37		25.40 (24.09)	18.01	
Yes	103	0.62 (0.45)	0.49		23.36 (18.69)	17.96	

*Mann–Whitney U test.

### Multivariate analysis

Table 7 presents a multivariate analysis of factors associated with ln(NO₂⁻) and ln(NO₃⁻) levels among Jordanian women, by region. For ln(NO₂⁻) in the whole sample, women residing in North Ghor had significantly higher levels than those in South Ghor (B = 0.59, 95% CI 0.42 to 0.77, *p* < 0.001). Self-reported hypertension was significantly associated with lower NO₂⁻ levels (B = − 0.44, 95% CI − 0.85 to − 0.04, *p* = 0.032), while eating green beans once a week or more remained significantly associated with higher NO₂⁻ levels (B = 0.20, 95% CI0.02 to 0.37, *p* = 0.031), independent of the other covariates in the model.

**Table 7:** Multivariate analysis of factors associated with ln(NO₂⁻) and ln(NO₃⁻) levels among Jordanian women, by region*

Dependent variable/region		Variable	B	95% confidence interval	*P*-value
Ln (NO₂⁻)					
Whole sample	Maternal age (years)	0.01	− 0.01	0.02	0.384
	Pre-pregnancy BMI (kg/m^2^)	0.00	− 0.01	0.01	0.995
	Hypertension (yes vs. no)	− 0.44	− 0.85	− 0.04	0.032
	Residency (North vs. South Ghor)	0.59	0.42	0.77	< 0.001
	Green beans ≥ 1/week (yes vs. no)	0.20	0.02	0.37	0.031
	Chard ≥ 1/week (yes vs. no)	− 0.08	− 0.32	0.15	0.478
	Radishes ≥ 1/week (yes vs. no)	− 0.04	− 0.21	0.14	0.678
	Anemia (yes vs. no)	0.08	− 0.09	0.25	0.369
	Smoking during pregnancy (yes vs. no)	0.14	− 0.16	0.44	0.367
	Municipal water for irrigation (yes vs. no)	0.10	− 0.11	0.30	0.346
	Employment in crop harvesting/collection (yes vs. no)	0.07	− 0.20	0.35	0.602
North Ghor	Maternal age (years)	0.01	− 0.01	0.03	0.262
	Pre-pregnancy BMI (kg/m^2^)	− 0.00	− 0.02	0.01	0.643
	Hypertension (yes vs. no)	− 0.66	− 1.18	− 0.14	0.013
	Green beans ≥ 1/week (yes vs. no)	0.24	0.02	0.47	0.032
	Chard ≥ 1/week (yes vs. no)	− 0.04	− 0.30	0.22	0.750
	Radishes ≥ 1/week (yes vs. no)	0.12	− 0.09	0.33	0.246
	Anemia (yes vs. no)	0.18	− 0.01	0.38	0.068
	Smoking during pregnancy (yes vs. no)	0.27	− 0.07	0.60	0.123
	Municipal water for irrigation (yes vs. no)	0.15	− 0.11	0.41	0.248
	Employment in crop harvesting/collection (yes vs. no)	0.10	− 0.18	0.38	0.487
South Ghor	Maternal age (years)	0.01	− 0.02	0.03	0.690
	Pre-pregnancy BMI (kg/m^2^)	0.00	− 0.02	0.03	0.729
	Hypertension (yes vs. no)	− 0.30	− 0.95	0.35	0.357
	Green beans ≥ 1/week (yes vs. no)	− 0.02	− 0.34	0.31	0.914
	Chard ≥ 1/week (yes vs. no)	− 0.17	− 0.65	0.31	0.481
	Radishes ≥ 1/week (yes vs. no)	− 0.29	− 0.59	0.01	0.057
	Anemia (yes vs. no)	− 0.09	− 0.42	0.24	0.582
	Smoking during pregnancy (yes vs. no)	− 0.12	− 0.72	0.48	0.704
	Municipal water for irrigation (yes vs. no)	0.06	− 0.28	0.40	0.734
	Employment in crop harvesting/collection (yes vs. no)	− 0.02	− 0.66	0.61	0.947
Ln (NO₃⁻)					
Whole sample	Maternal age (years)	0.00	− 0.01	0.02	0.685
	Pre-pregnancy BMI (kg/m^2^)	− 0.01	− 0.02	0.00	0.176
	Hypertension (yes vs. no)	0.19	− 0.17	0.56	0.300
	Residency (North vs. South Ghor)	0.16	− 0.00	0.32	0.056
	Green beans ≥ 1/week (yes vs. no)	− 0.06	− 0.22	0.10	0.485
	Chard ≥ 1/week (yes vs. no)	0.06	− 0.15	0.27	0.559
	Radishes ≥ 1/week (yes vs. no)	0.01	− 0.15	0.17	0.890
	Anemia (yes vs. no)	− 0.05	− 0.20	0.11	0.572
	Smoking during pregnancy (yes vs. no)	− 0.42	− 0.70	− 0.14	0.003
	Municipal water for irrigation (yes vs. no)	− 0.18	− 0.37	0.00	0.055
	Employment in crop harvesting/collection (yes vs. no)	0.25	− 0.00	0.50	0.053
North Ghor	Maternal age (years)	0.00	− 0.01	0.02	0.555
	Pre-pregnancy BMI (kg/m^2^)	− 0.01	− 0.02	0.01	0.334
	Hypertension (yes vs. no)	0.11	− 0.30	0.52	0.600
	Green beans ≥ 1/week (yes vs. no)	− 0.09	− 0.26	0.09	0.328
	Chard ≥ 1/week (yes vs. no)	0.14	− 0.07	0.34	0.182
	Radishes ≥ 1/week (yes vs. no)	0.06	− 0.10	0.23	0.450
	Anemia (yes vs. no)	0.05	− 0.11	0.20	0.528
	Smoking during pregnancy (yes vs. no)	− 0.11	− 0.38	0.15	0.400
	Municipal water for irrigation (yes vs. no)	− 0.01	− 0.22	0.19	0.895
	Employment in crop harvesting/collection (yes vs. no)	0.06	− 0.17	0.28	0.616
South Ghor	Maternal age (years)	− 0.00	− 0.03	0.02	0.709
	Pre-pregnancy BMI (kg/m^2^)	− 0.01	− 0.03	0.01	0.346
	Hypertension (yes vs. no)	0.14	− 0.51	0.78	0.675
	Green beans ≥ 1/week (yes vs. no)	− 0.07	− 0.39	0.25	0.665
	Chard ≥ 1/week (yes vs. no)	0.09	− 0.39	0.56	0.721
	Radishes ≥ 1/week (yes vs. no)	− 0.06	− 0.36	0.23	0.669
	Anemia (yes vs. no)	− 0.18	− 0.50	0.15	0.289
	Smoking during pregnancy (yes vs. no)	− 0.89	− 1.49	− 0.30	0.004
	Municipal water for irrigation (yes vs. no)	− 0.28	− 0.62	0.07	0.113
	Employment in crop harvesting/collection (yes vs. no)	0.73	0.10	1.37	0.024

*All models used a forced-entry (knowledge-based) approach with the same covariate set entered simultaneously: maternal age, pre-pregnancy BMI, hypertension status, anemia, and smoking during pregnancy were forced as core covariates in every model; region (North Ghor vs. South Ghor) was additionally forced in the whole-sample models; and green bean, chard, and radish consumption (≥ 1/week vs. less), municipal water use for irrigation, and employment in crop harvesting/collection were entered simultaneously as the dietary/environmental exposures of interest.

Within North Ghor, hypertension was significantly associated with lower NO₂⁻ levels (B = − 0.66, 95% CI − 1.18 to − 0.14, *p* = 0.013), and green bean consumption remained associated with higher levels (B = 0.24, 95% CI 0.02 to 0.47, *p* = 0.032). In South Ghor, no covariate reached statistical significance in the forced-entry model; radish consumption showed a borderline inverse association with NO₂⁻ levels (B = − 0.29, 95% CI − 0.59 to 0.01, *p* = 0.057), consistent in direction with the unadjusted comparison in Table 6 but attenuated below significance once the full covariate set was accounted for.

Regarding ln(NO₃⁻) levels, in the whole-sample model, the only covariate significantly associated with NO₃⁻ levels was smoking during pregnancy, with smokers having lower levels than non-smokers (B = − 0.42, 95% CI − 0.70 to − 0.14, *p* = 0.003). Within North Ghor, no covariate reached significance, including chard consumption (B = 0.14, 95% CI − 0.07 to 0.34, *p* = 0.182). In South Ghor, smoking during pregnancy (B = − 0.89, 95% CI − 1.49 to − 0.30, *p* = 0.004) and employment in crop harvesting/collection (B = 0.73, 95% CI 0.10 to 1.37, *p* = 0.024) were significantly associated with NO₃⁻ levels.

## Discussion

### Regional variation in serum NO₂⁻ and NO₃⁻ levels

Women residing in North Ghor had significantly higher serum NO₂⁻ levels than women in South Ghor, while serum NO₃⁻ levels did not differ significantly between the two regions; this regional contrast in NO₂⁻ persisted after adjusting for maternal age, BMI, hypertension, anemia, smoking, and the dietary and environmental exposures examined in this study. Similar short-distance regional contrasts in nitrate/nitrite biomarkers have been reported in other agricultural settings where dietary habits, rather than climate or water infrastructure, vary markedly between neighboring communities^5–9^. North and South Ghor differed substantially in reported vegetable consumption, with North Ghor reporting higher intake of cabbage, fenugreek, celery, radishes, turnips, and chard. Water-source variables did not differ as consistently between regions and were not significant predictors of NO₂⁻ levels in the adjusted model. This pattern suggests that regional dietary habits, rather than water source, are the more likely driver of the NO₂⁻ difference observed between regions.

### Dietary patterns and NO₂⁻/NO₃⁻ exposure

Eating green beans once a week or more was independently associated with higher serum NO₂⁻ levels in the whole sample, as well as in North Ghor in the adjusted model. This is consistent with the broader literature on vegetable-derived NO₃⁻/NO₂⁻ exposure, in which leafy and legume vegetables are established dietary contributors to systemic nitrate/nitrite levels^14–21^. Dietary NO₃⁻ is absorbed in the upper gastrointestinal tract and partially reduced to NO₂⁻ via the enterosalivary pathway^1,18^, providing a plausible mechanism for the green bean–NO₂⁻ association. Radish consumption in South Ghor showed an inverse, borderline association with serum NO₂⁻ levels in the unadjusted comparison that was attenuated after adjustment (*p* = 0.057); given this attenuation and the absence of a clear mechanistic explanation, we treat this as a hypothesis-generating observation rather than a confirmed finding.

### Hypertension and serum NO₂⁻ levels

Hypertension was independently associated with significantly lower serum NO₂⁻ levels, both in the whole sample (B = − 0.44, 95% CI − 0.85 to − 0.04, *p* = 0.032) and in North Ghor (B = − 0.66, 95% CI − 1.18 to − 0.14, *p* = 0.013), after adjustment for region, age, BMI, anemia, smoking, and dietary/environmental exposures. This direction is consistent with a study of pregnant women that reported lower serum nitrite in those with chronic hypertension (19.6 µmol/L) than in normotensive controls (25.4 µmol/L), in contrast to the higher nitrite levels reported among women with preeclampsia in the same and other studies, suggesting that chronic hypertension and preeclampsia may be associated with NO metabolism in opposite directions^22^. Lower NO₂⁻ in hypertensive women may reflect reduced NO bioavailability or endothelial dysfunction, consistent with impaired NO-mediated vasodilation contributing to elevated blood pressure^14,23^. We had hypothesized that dietary modification (e.g., reduced salt or processed-meat intake commonly recommended for hypertensive patients) might also contribute to lower NO₂⁻ intake in this group; however, we did not collect data on sodium or processed-meat intake specifically, so this remains an untested hypothesis rather than a finding of this study.

### Water source and environmental exposure

The use of non-municipal groundwater for irrigation in South Ghor was not significantly associated with serum NO₃⁻ levels. This contrasts with a substantial literature identifying groundwater and irrigation water as a major NO₃⁻ exposure route in agricultural regions, including elsewhere in the MENA region^1,3,4,7–9^. In this population, dietary and occupational pathways may contribute more directly to serum NO₃⁻/NO₂⁻ levels than the water source. However, our study focused on serum NO₃⁻/NO₂⁻ measurements, and the water source was assessed only by self-reported category (e.g., municipal, well, filtered) rather than by direct chemical measurement of NO₃⁻/NO₂⁻ concentrations in the water itself, and no vegetable or soil samples were analyzed.

### Occupational exposure and smoking

In South Ghor, employment in crop harvesting/collection was independently associated with higher serum NO₃⁻ levels (B = 0.73, 95% CI 0.10 to 1.37, *p* = 0.024), and smoking during pregnancy was independently associated with lower serum NO₃⁻ levels both in the whole sample (B = − 0.42, 95% CI − 0.70 to − 0.14, *p* = 0.003) and in South Ghor (B = − 0.89, 95% CI − 1.49 to − 0.30, *p* = 0.004). Occupational handling of fertilized crops and produce has been linked to elevated nitrate body burden among agricultural workers in other settings, consistent with direct dermal, inhalational, or hand-to-mouth contact during harvesting. The crop-harvesting finding is biologically plausible as a direct, non-dietary occupational exposure pathway distinct from food consumption. An inverse association between smoking and circulating NO₃⁻/NO₂⁻ is less commonly reported in the general population literature, where smoking is more often linked to altered NO synthesis through oxidative stress pathways. The inverse smoking association should be interpreted cautiously: only 26 of 346 participants (7.4%) reported smoking during pregnancy; the finding could reflect residual confounding by dietary patterns not fully captured by the vegetables assessed in this questionnaire. We present this as a novel, hypothesis-generating observation that warrants replication in a larger sample with more detailed dietary and smoking data, rather than as an established finding.

### Socio-demographic factors

Maternal age, education, and occupation were not significantly associated with serum NO₃⁻ or NO₂⁻ levels in either the bivariate comparisons or the adjusted multivariable models. This pattern suggests that, within this rural population, region, diet, and occupational exposure to crop harvesting are more relevant determinants of NO₃⁻/NO₂⁻ exposure than broad socio-demographic characteristics.

### Comparison with other populations

The median serum NO₃⁻ level observed in this study (19.13 µM) falls within the 95% reference range reported for serum NO₃⁻ + NO₂⁻ (NOx) in a large cohort of healthy, non-smoking adults from Iran (10.1–65.6 µmol/L in women)^24^, and is broadly comparable to plasma nitrate concentrations reported among healthy adults in placebo/control conditions^25^. This suggests that, despite residing in agricultural communities, most participants in this study were not experiencing markedly elevated NO₃⁻ burden relative to these general population reference values.

The median serum NO₂⁻ level observed (0.63 µM) aligns with ranges reported in multiple adult cohorts, though it falls below levels reported in an older report on pregnant women^22^. We cannot fully account for this difference, but it may partly reflect methodological factors such as the additional serum ultrafiltration step used in this study, differences in sample handling and storage, or assay calibration; this underscores the difficulty of directly comparing NO₂⁻ values across studies using different protocols, and we caution against over-interpreting absolute NO₂⁻ levels in cross-study comparisons.

### Public health implications

These findings carry public health implications for rural, agricultural settings where dietary habits and occupational activities are closely tied to local food production. Interventions to reduce NO₃⁻/NO₂⁻ exposure should consider regional dietary patterns alongside occupational protective measures (e.g., gloves and hand hygiene during crop harvesting), particularly for pregnant women who continue agricultural work. Public awareness about dietary sources of NO₃⁻/NO₂⁻ may help communities make informed choices, though we emphasize that the moderate NO₃⁻ levels observed relative to general population reference ranges do not, on their own, indicate an urgent population-level health risk in this cohort.

### Limitations

This study has several limitations. Dietary and water source intake quantity, farming activity, and medical history were self-reported, and due to financial and logistical barriers the NO₃⁻/NO₂⁻ levels from these sources were not measured. We used the Griess assay for analyses of NO₃⁻/NO₂⁻ which may be affected by interference from other nitrogenous compounds and was not cross-validated using more specific methods such as HPLC or mass spectrometry. The cross-sectional design limits causal inference, and serum NO₃⁻/NO₂⁻ reflects recent rather than cumulative exposure during pregnancy. Finally, excluding women with adverse pregnancy outcomes may limit generalizability to higher-risk pregnancies, and the absence of urinary or salivary NO₃⁻/NO₂⁻ measurements limited characterization of total nitrate/nitrite metabolism.

## Conclusion

This study highlights the influence of region-specific dietary behaviors and, in South Ghor, occupational exposure to crop harvesting, in shaping serum NO₃⁻ and NO₂⁻ levels among women in rural Jordan. Hypertension and green bean consumption were robust predictors of serum NO₂⁻, while smoking during pregnancy and crop-harvesting work emerged as the more robust predictors of serum NO₃⁻. Effectively addressing NO₃⁻ and NO₂⁻ exposure in this setting will likely require a combination of dietary guidance, occupational hygiene measures for women engaged in agricultural work, and further research incorporating direct environmental sampling and biomarker validation.

## Data Availability

Data is available upon reasonable request by investigators, but cannot be made publicly available because of the risk to privacy and confidentiality of sensitive health data among this high-risk population. The participants did not authorize the authors to make their data publicly available or to be used for other aims.

## References

[1] Ward, M. H. et al. Drinking water nitrate and human health: An updated review. *Int J Environ Res Public Health.* **15**(7), 1557. 10.3390/ijerph15071557 (2018). PMC6068531 30041450

[2] Brkić, D. et al. Nitrate in leafy green vegetables and estimated intake. *Afr. J. Tradit. Complement. Altern. Med.* **14**(3), 31–41. 10.21010/ajtcam.v14i3.4 (2017). PMC5412236 28480414

[3] Taneja, P., Labhasetwar, P., Nagarnaik, P. & Ensink, J. H. J. The risk of cancer as a result of elevated levels of nitrate in drinking water and vegetables in Central India. *J. Water Health* **15**(4), 602–614. 10.2166/wh.2017.283 (2017). 28771157

[4] Brender, J. D. et al. Prenatal nitrate intake from drinking water and selected birth defects in offspring of participants in the National Birth Defects Prevention Study. *Environ. Health Perspect.* **121**(9), 1083–1089. 10.1289/ehp.1206249 (2013). PMC3764078 23771435

[5] Al-Addous, M., Bdour, M., Alnaief, M., Rabaiah, S. & Schweimanns, N. Water resources in Jordan: A review of current challenges and future opportunities. *Water* **15**(21), 3729 (2023).

[6] van Grinsven, H. J. M. et al. Management, regulation and environmental impacts of nitrogen fertilization in northwestern Europe under the Nitrates Directive; a benchmark study. *Biogeosciences* **9**, 5143–5160. 10.5194/bg-9-5143-2012 (2012).

[7] Hejaz, B., Al-Khatib, I. A. & Mahmoud, N. Domestic groundwater quality in the northern governorates of the West Bank, Palestine. *J. Environ. Public Health* 10.1155/2020/6894805 (2020). PMC7142351 32300368

[8] Troudi, N., Hamzaoui-Azaza, F., Tzoraki, O., Melki, F. & Zammouri, M. Assessment of groundwater quality for drinking purpose with special emphasis on salinity and nitrate contamination in the shallow aquifer of Guenniche (Northern Tunisia). *Environ. Monit. Assess.* **192**(10), 641. 10.1007/s10661-020-08572-4 (2020). 32929589

[9] Zakhem, B. A. & Hafez, R. Hydrochemical, isotopic and statistical characteristics of groundwater nitrate pollution in Damascus Oasis (Syria). *Environ. Earth Sci.* **74**(4), 2781–2797. 10.1007/s12665-015-4258-1 (2015).

[10] Moncada, S., Palmer, R. M. J. & Higgs, E. A. Nitric oxide: Physiology, pathophysiology, and pharmacology. *Pharmacol. Rev.* **43**(2), 109–142 (1991). 1852778

[11] Mirvish, S. S. Role of N-nitroso compounds (NOC) and N-nitrosation in etiology of gastric, esophageal, nasopharyngeal and bladder cancer and contribution to cancer of known exposures to NOC. *Cancer Lett.* **93**(1), 17–48. 10.1016/0304-3835(95)03786-V (1995). 7600541

[12] Lin, L. et al. Nitrate contamination in drinking water and adverse reproductive and birth outcomes: A systematic review and meta-analysis. *Sci. Rep.* **13**(1), 563. 10.1038/s41598-022-27345-x (2023). PMC9834225 36631499

[13] Vuong, A. M. et al. Prenatal exposure to nitrosatable drugs, dietary intake of nitrites, and preterm birth. *Am. J. Epidemiol.* **183**(7), 634–642. 10.1093/aje/kwv250 (2016). 26953287

[14] Bahadoran, Z. et al. NitrateNO₃⁻ and nitriteNO₂⁻ content of vegetables, fruits, grains, legumes, dairy products, meats and processed meats. *J. Food Compos. Anal.* **51**, 93–105. 10.1016/j.jfca.2016.06.006 (2016).

[15] Czech, A., Pawlik, M. & Rusinek, E. Contents of heavy metals, nitrates, and nitrites in cabbage. Pol. J. Environ. Stud. **21** (2012).

[16] Ko, Y. M., Park, J. H. & Yoon, K. S. Nitrite formation from vegetable sources and its use as a preservative in cooked sausage. *J. Sci. Food Agric.* **97**, 1774–1783 (2017). 10.1002/jsfa.7974 27469979

[17] Wang, Z. & Shao, Y. Effects of microbial diversity on nitrite concentration in pao cai, a naturally fermented cabbage product from China. *Food Microbiol.* **72**, 185–192 (2018). 10.1016/j.fm.2017.12.003 29407396

[18] Hord, N. G., Tang, Y. & Bryan, N. S. Food sources of nitrates and nitrites: The physiologic context for potential health benefits. *Am. J. Clin. Nutr.* **90**(1), 1–10. 10.3945/ajcn.2008.27131 (2009). 19439460

[19] Iammarino, M. et al. Determination of nitrate and nitrite in Swiss chard (*Beta vulgaris* L. subsp. *vulgaris*) and wild rocket (*Diplotaxis tenuifolia* (L.) DC.) and food safety evaluations. Foods 10.3390/foods11172571 (2022). PMC9455605 36076757

[20] Schopfer, B. et al. Red beet and Swiss chard juice extract as natural nitrate sources for the production of alternatively-cured emulsion-type sausages. *Meat Sci.* **188**, 108780. 10.1016/j.meatsci.2022.108780 (2022). 35279476

[21] Kelany, M. A., Mohamed, R. G., El-Shikh, K. A. & Sorour, M. A. Effects of refrigeration and freezing on nitrate, nitrite, and oxalate levels in green beans and peas. *J. Sohag Agrisci. (JSAS)* **9**, 138–147. 10.21608/jsasj.2024.402772 (2024).

[22] Bartha, J. L. et al. Maternal serum nitric oxide levels associated with biochemical and clinical parameters in hypertension in pregnancy. *Eur. J. Obstet. Gynecol. Reprod. Biol.* **82**(2), 201–207. 10.1016/s0301-2115(98)00234-6 (1999). 10206416

[23] Norouzzadeh, M., Rashedi, M. H., Payandeh, N., Harijani, A. M. & Shahinfar, H. The effects of dietary nitrate on blood pressure and vascular health: An umbrella review and updated meta-analysis and meta-regression. *J. Funct. Foods* **114**, 106082. 10.1016/j.jff.2024.106082 (2024).

[24] Ghasemi, A., Zahediasl, S. & Azizi, F. Reference values for serum nitric oxide metabolites in an adult population. *Clin Biochem.* **43**(1–2), 89–94. 10.1016/j.clinbiochem.2009.09.008 (2010). 19782059

[25] Ho, X. L. & Loke, W. M. Dietary plant sterols supplementation increases in vivo nitrite and nitrate production in healthy adults: A randomized, controlled study. *J. Food Sci.* **82**(7), 1750–1756. 10.1111/1750-3841.13752 (2017). PMC5601184 28708316

